# Implications of Habitual Alcohol Intake With the Prognostic Significance of Mean Corpuscular Volume in Stage II-III Colorectal Cancer

**DOI:** 10.3389/fonc.2021.681406

**Published:** 2021-06-14

**Authors:** Qi Liu, Yufei Yang, Xinxiang Li, Sheng Zhang

**Affiliations:** ^1^ Department of Colorectal Surgery, Fudan University Shanghai Cancer Center, Shanghai, China; ^2^ Department of Oncology, Shanghai Medical College, Fudan University, Shanghai, China; ^3^ Guangdong Lung Cancer Institute, Guangdong Provincial People’s Hospital, Guangdong Academy of Medical Sciences, Guangzhou, China

**Keywords:** alcohol intake, mean corpuscular volume, prognostic, stage II-III, colorectal cancer

## Abstract

**Objective:**

To elucidate the prognostic significance of mean corpuscular volume (MCV), with implications of habitual alcohol intake in stage II-III colorectal cancer (CRC).

**Background:**

MCV had the potential to become an ideal prognostic biomarker and be put into clinical application. Few studies, however, have explored whether habitual alcohol intake which greatly increased the value of MCV would affect the prognostic role of MCV.

**Methods:**

Eligible patients were identified from the CRC database of Fudan University Shanghai Cancer Center (FUSCC) between January 2012 and December 2013. Survival analyses were constructed using the Kaplan–Meier method to evaluate the survival time distribution, and the log-rank test was used to determine the survival differences. Univariate and multivariate Cox proportional hazard models were built to calculate the hazard ratios of different prognostic factors.

**Results:**

A total of 694 patients diagnosed with stage II-III CRC between January 2012 and December 2013 were identified from FUSCC. Low pretreatment MCV was independently associated with 72.0% increased risk of overall mortality compared with normal MCV (HR = 1.720, 95%CI =1.028-2.876, P =0.039, using normal MCV as the reference). In patients with habitual alcohol intake, however, pretreatment MCV positively correlated with the mortality (P = 0.02) and tumor recurrence (P = 0.002) after adjusting for other known prognostic factors.

**Conclusions:**

In CRC patients without habitual alcohol intake, low (<80 fL) level of pretreatment MCV was a predictor of poor prognosis. In patients with habitual alcohol intake, however, pretreatment MCV showed the opposite prognostic role, which would elicit many fundamental studies to elucidate the mechanisms behind.

## Introduction

Colorectal cancer (CRC) was one of the most commonly diagnosed malignances worldwide (1). Among them, stage II (T3-4N0M0) and stage III (TanyN1-2M0) diseases accounted for a vast majority (2, 3). Despite the significant improvements of oncologic outcomes in stage II-III CRC due to the development of surgery techniques and adjuvant therapy over the past decades, 30% of stage II and 50-60% of stage III CRC patients were reported to experience a recurrence within 5 years after the operations (4).

Over the past decades, researchers were looking for new biomarkers related to cancer incidence, mortality and oncologic outcomes (5, 6). However, reliable, low-cost and easily accessible biomarkers that can be optimally put into a real clinical application were still rare.

As a measure of the average volume of a red blood cell, mean corpuscular volume (MCV) was related to the prognosis of liver cancer (7), esophageal cancer (8) and adenocarcinomas of the gastroesophageal junction (9). Interestingly enough, MCV was also reported to be associated with the risk of colorectal adenoma (10), advanced CRC (11) and response to chemotherapy in CRC (12, 13), suggesting MCV had the potential to be an ideal biomarker and be put into clinical application. In particular, previous study revealed that high MCV value may be used as an index of the risk of colorectal adenomas (10), but a recent research reported decreased MCV was an independent predictor for the detection of advanced colorectal cancer (11), indicating that the clinical role of MCV in colorectal cancer was still uncertain.

Alcohol drinking, which was an important health and social problem worldwide, was a significant cause of higher MCV (9, 14, 15). Alcohol drinking was one of the global health priorities, however, to the best of our knowledge, no previous studies have investigated the prognostic value of MCV in CRC patients with habitual alcohol intake (16). Therefore, we conducted this study to elucidate the prognostic significance of MCV with implications of habitual alcohol intake in CRC.

## Methods

### Patient Selection

In the present study, we identified patients meeting the following criteria from the CRC database of Fudan University Shanghai Cancer Center (FUSCC) between January 2012 and December 2013: (1) diagnosed with stages II or III CRC by histopathology; (2) without neoadjuvant treatment; (3) underwent curative surgery without positive surgical margin; (4) adenocarcinoma; (5) with the information of pretreatment MCV and carcinoembryonic antigen (CEA); (6) without history of gastrectomy, upper aerodigestive tract cancer, recent bleeding or anemia; (7) with complete relevant demographic and clinicopathologic data. Nine patients (1.3%) with high pretreatment MCV (>100fL) were also excluded from the cohort because of the small sample size (**Figure S1**). Eligible patients were divided into two groups according to the standard value of pretreatment MCV: normal-MCV group (80–100fL) and low-MCV (<80fL) group. We then extracted the demographic and clinicopathological characteristics of patients from FUSCC database including the information of pretreatment MCV and CEA from blood routine examination (all the blood samples were obtained from patients within 3 days prior to the radical resection). In our center, 5-Fu-based adjuvant chemotherapy was recommended for both high-risk pathological stage II diseases and stage III diseases. The information of alcohol intake was extracted from of personal history, those with habitual alcohol intake recently were identified. This study was approved by the Ethical Committee and Institutional Review Board of FUSCC.

### Statistical Analyses

In this study, Pearson’s chi-squared test was used to compare clinicopathological and demographic characteristics according to the levels of pretreatment MCV. Survival analyses were conducted using the Kaplan–Meier method to evaluate the survival time distribution, and the log-rank test was used to determine the univariate survival difference. Univariate and multivariate Cox proportional hazard models were constructed to calculate the hazard ratios of prognostic factors, including tumor grade (high/moderate or low), habitual alcohol intake (yes or no), vascular invasion (yes or no), nerve invasion (yes or no), serum CEA levels (high or low), gender (male or female), age at diagnosis (years), tumor location (rectum or colon), postoperative complications (yes or no), stage (II or III), adjuvant treatment (yes or no), and No. of lymph nodes retrieved (<12 or ≥12). Only the clinicopathological characteristics that showed prognostic significance (log-rank, P < 0.20) in the univariate Cox analyses were included into the multivariate Cox analyses. A variable with two-sided *P <*0.05 was considered statistically significant. Statistical analyses in the present study were carried out using the SPSS version 22 (IBM Corporation, Armonk, NY, USA).

## Results

### Clinical Characteristics of Patients From FUSCC

A total of 694 patients diagnosed with stage II-III CRC between January 2012 and December 2013 were identified from FUSCC. The median follow-up time among the whole cohort was 68 months. Among them, 409 (58.9%) patients were men and 285 (41.1%) patients were women; 81 (11.7%) patients were associated with low levels of pretreatment MCV and 613 (88.3%) patients were associated with normal levels of pretreatment MCV; 122 (17.6%) patients had habitual alcohol intake and 572 (82.4%) patients not; the median age at diagnosis was 60 years; 313 (45.1%) patients were diagnosed with colon cancer and 381 (54.9%) patients were diagnosed with rectal cancer; 331 (47.7%) patients were with stage II disease and 363 (52.3%) patients were with stage III disease. The baseline characteristics according to the pretreatment MCV levels were shown in **Table 1**. Low MCV was significantly associated with low tumor grade, female and colon cancer (P < 0.05).

**Table 1:** Baseline characteristics of the of the overall cohort by the levels of pretreatment MCV.

Characteristics	No. of Patients (%)		P
	Low MCV (fl) (n=81)	Normal MCV (fl) (n=613)	
**Tumor grade**			0.004
** High/Moderate**	48 (59.3)	456 (74.4)	
** Low**	33 (40.7)	157 (25.6)	
**Habitual alcohol intake**			0.700
** No**	68 (84.0)	504 (82.2)	
** Yes**	13 (16.0)	109 (17.8)	
**Vascular invasion**			0.549
** No**	63 (77.8)	458 (74.7)	
** Yes**	18 (22.2)	155 (25.3)	
**Nerve invasion**			0.802
** No**	65 (80.2)	499 (81.4)	
** Yes**	16 (19.8)	114 (18.6)	
**Pretreatment CEA levels**			0.066
** Normal**	39 (48.1)	361 (58.9)	
** High**	42 (51.9)	252 (41.1)	
**Gender**			0.036
** Male**	39 (48.1)	370 (60.4)	
** Female**	42 (51.9)	243 (39.6)	
**Age at diagnosis (years)**			0.920
** <65**	52 (64.2)	397 (64.8)	
** ≥65**	29 (35.8)	216 (35.2)	
**Tumor location**			<0.001
** Rectum**	14 (17.3)	299 (48.8)	
** Colon**	67 (82.7)	314 (51.2)	
**Postoperative complications**			0.523
** No**	76 (93.8)	585 (95.4)	
** Yes**	5 (6.2)	28 (4.6)	
**Stage**			0.204
** II**	44 (54.3)	287 (46.8)	
** III**	37 (45.7)	326 (53.2)	
**Adjuvant treatment**			0.184
** No**	22 (27.2)	127 (20.7)	
** Yes**	59 (72.8)	486 (79.3)	
**No. of lymph nodes retrieved**			0.153
** <12**	5 (6.2)	70 (11.4)	
** ≥12**	76 (93.8)	543 (88.6)	

### Low MCV Was Associated With Worse Overall Survival in CRC

**Figure S2** showed the result of Kaplan-Meier OS analysis according to the pretreatment MCV levels. Compared with normal MCV, low MCV was significantly associated with reduced 5-year OS rate (87.3% *vs*. 76.4%, P < 0.0077). In addition, we also conducted univariate and multivariate Cox regression analyses to evaluate the prognostic value of clinicopathologic factors including MCV status (**Table 2**). In univariate analysis, low MCV was associated with 94.7% increased risk of overall mortality compared with normal MCV (HR = 1.947, 95%CI =1.181-3.211, P =0.009, using normal MCV as the reference; **Table 2**). The univariate analysis produced nine prognostic characteristics including MCV status, habitual alcohol intake, tumor grade, vascular invasion, nerve invasion, pretreatment CEA levels, age at diagnosis, tumor stage and the receipt of adjuvant treatment, which were included into multivariate analyses. It was shown that pretreatment MCV was also an independent prognostic factor and low pretreatment MCV was independently associated with 72.0% increased risk of overall mortality compared with normal level of MCV (HR = 1.720, 95%CI =1.028-2.876, P =0.039, using normal level of MCV as the reference; **Table 2**). In addition, it was also found that patients with habitual alcohol were independently associated with 75.4% increased risk of overall mortality compared with patients not (HR = 1.754, 95%CI =1.093-2.816, P =0.020, using without habitual alcohol intake as the reference; **Table 2**).

**Table 2:** Univariate and multivariate Cox regression analyses for OS in the whole cohort.

Characteristics	Univariate analyses		Multivariate analyses	
	HR (95%CI)	P value	HR (95%CI)	P value
**Pretreatment MCV (fl)**		0.009		0.039
** 80-100**	1		1	
** <80**	1.947 (1.181-3.211)		1.720 (1.028-2.876)	
**Habitual alcohol intake**		0.104		0.020
** No**	1		1	
** Yes**	1.472 (0.923-2.347)		1.754 (1.093-2.816)	
**Tumor grade**		<0.001		0.002
** High/Moderate**	1		1	
** Low**	2.372 (1.594-3.529)		1.940 (1.270-2.963)	
**Vascular invasion**		0.002		0.750
** No**	1		1	
** Yes**	1.895 (1.261-2.847)		0.928 (0.586-1.470)	
**Nerve invasion**		<0.001		0.001
** No**	1		1	
** Yes**	2.536 (1.675-3.840)		2.084 (1.351-3.216)	
**Pretreatment CEA levels**		0.001		0.005
** Normal**	1		1	
** High**	2.032 (1.362-3.032)		1.772 (1.186-2.647)	
**Gender**		0.603		
** Male**	1			
** Female**	1.111 (0.747-1.653)			
**Age at diagnosis (years)**		0.002		0.003
** <65**	1		1	
** ≥65**	1.893 (1.276-2.807)		1.890 (1.237-2.887)	
**Tumor location**		0.433		
** Rectum**	1			
** Colon**	1.174 (0.787-1.751)			
**Postoperative complications**		0.221		
** No**	1			
** Yes**	1.617 (0.749-3.488)			
**Stage**		<0.001		<0.001
** II**	1		1	
** III**	3.234 (2.028-5.157)		3.619 (2.132-6.145)	
**Adjuvant treatment**		0.112		0.008
** No**	1		1	
** Yes**	0.698 (0.449-1.087)		0.511 (0.311-0.839)	
**No. of lymph nodes retrieved**		0.629		
** <12**	1			
** ≥12**	0.862 (0.471-1.576)			

**Figure 1:**
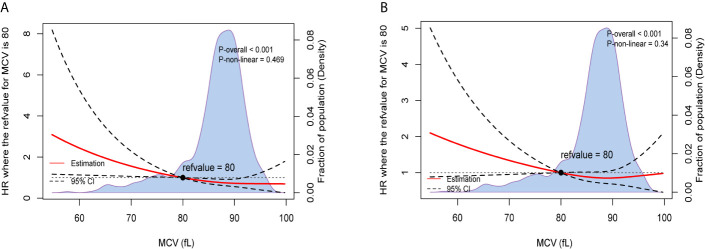
Preoperative MCV and the corresponding hazard ratios on a continuous scale, including **(A)**. OS after adjusting for other prognostic factors; **(B)**. RFS after adjusting for other prognostic factors. Analyses were carried out using restricted cubic splines, with hazard ratios and 95% confidence intervals from multivariate Cox proportional hazards regression. The pretreatment MCV of 80fL was chosen as the reference. The purple area indicated the distribution of concentration of the pretreatment MCV.

After adjusting for other prognostic factors, we also used restricted cubic splines to show the preoperative MCV levels and the corresponding HRs of OS and recurrence-free survival (RFS) on a continuous scale (**Figures 1A, B**). Similarly, it was clear that low level of MCV negatively correlated with the mortality and tumor recurrence after adjusting for other prognostic factors.

### Prognostic Role of Pretreatment MCV With the Complications of Habitual Alcohol Intake

**Figure 2:**
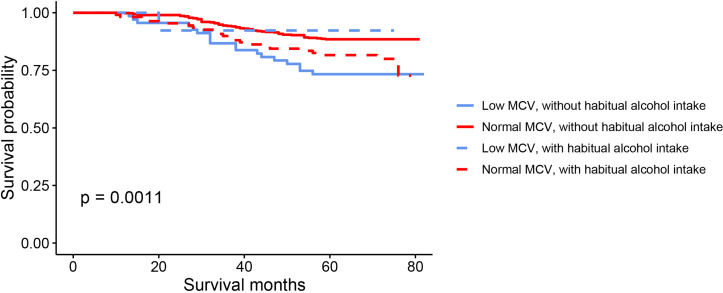
Kaplan–Meier OS curves according to the levels of pretreatment MCV with the implications of habitual alcohol intake.

**Figure 3:**
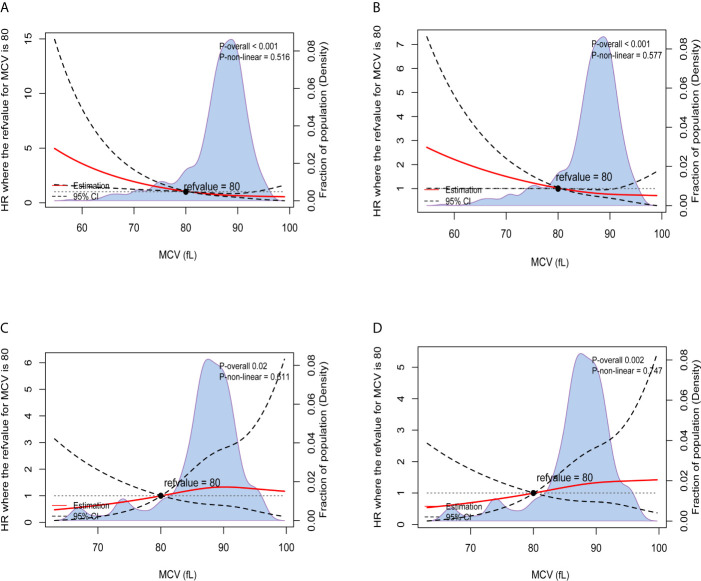
Preoperative MCV and the corresponding hazard ratios on a continuous scale with the implications of habitual alcohol intake, including **(A)**. OS after adjusting for other prognostic factors, without habitual alcohol intake; **(B)**. RFS after adjusting for other prognostic factors, without habitual alcohol intake; **(C)**. OS after adjusting for other prognostic factors, with habitual alcohol intake; **(D)**. RFS after adjusting for other prognostic factors, with habitual alcohol intake. Analyses were conducted using restricted cubic splines, with hazard ratios and 95% confidence intervals from multivariate Cox proportional hazards regression. The pretreatment MCV of 80fL was selected as the reference. The purple area indicated the distribution of concentration of the pretreatment MCV.

The results of Kaplan-Meier OS analysis according to pretreatment MCV levels with the implications of habitual alcohol intake were shown in **Figure 2**. In patients without habitual alcohol intake, compared with normal level of MCV, low level of MCV was significantly associated with worse reduced 5-year OS rate (88.5% VS. 73.3%, P < 0.001); in patients with habitual alcohol intake, however, low level of MCV (92.3%) had better 5-year OS rate compared with normal level of MCV (81.6%), while the survival difference did not achieve statistical significance (P = 0.342). Using restricted cubic splines, we then showed preoperative MCV levels and the corresponding HRs of OS and RFS on a continuous scale with the complications of habitual alcohol intake (**Figures 3A–D**). In patients without habitual alcohol intake, pretreatment MCV still negatively correlated with the mortality (**Figure 3A**, P < 0.001) and tumor recurrence (**Figure 3B**, P < 0.001) after adjusting for other prognostic factors; in patients with habitual alcohol intake, however, pretreatment MCV positively correlated with the mortality (**Figure 3C**, P = 0.02) and tumor recurrence (**Figure 3D**, P = 0.002) after adjusting for other prognostic factors, showing the opposite prognostic role of pretreatment MCV compared with patients without habitual alcohol intake.

The results of multivariate Cox regression analyses of OS in CRC patients without habitual alcohol intake also showed that pretreatment MCV was an independent prognostic factor and low pretreatment MCV was independently associated with 133.0% increased risk of overall mortality compared with normal level of MCV (HR = 2.330, 95%CI =1.350-4.020, P = 0.002, using normal level of MCV as the reference; **Table 3**), meaning that the poor prognostic role was even more pronounced in CRC patients without habitual alcohol intake than in the whole cohort.

**Table 3:** Multivariate Cox regression analyses for OS in patients without habitual alcohol intake.

Characteristics	Multivariate analyses	
	HR (95%CI)	P value
**Pretreatment MCV (fl)**		0.002
** 80-100**	1	
** <80**	2.330 (1.350-4.020)	
**Tumor grade**		0.001
** High/Moderate**	1	
** Low**	2.321 (1.441-3.739)	
**Vascular invasion**		0.574
** No**	1	
** Yes**	0.860 (0.507-1.458)	
**Nerve invasion**		0.017
** No**	1	
** Yes**	1.842 (1.118-3.035)	
**Pretreatment CEA levels**		0.006
** Normal**	1	
** High**	1.935 (1.214-3.083)	
**Age at diagnosis (years)**		0.003
** <65**	1	
** ≥65**	2.062 (1.273-3.342)	
**Stage**		<0.001
** II**	1	
** III**	3.627 (1.992-6.603)	
**Adjuvant treatment**		0.054
** No**	1	
** Yes**	0.568 (0.319-1.010)	

## Discussion

In the present study, 81 (11.7%) patients were associated with low levels of MCV, and 613 (88.3%) patients were associated with normal levels of MCV. It was found that low (<80 fL) level of pretreatment MCV was a poor prognostic feature in CRC, both in univariate and multivariate analyses. And low pretreatment MCV was independently associated with 72.0% increased risk of overall mortality compared with normal level of MCV; in CRC patients without habitual alcohol intake, furtherly, results of multivariate Cox analyses showed that this number increased to 133.0% compared with CRC patients with normal level of MCV, meaning that the poor prognostic role of low pretreatment MCV was even more pronounced than in the whole cohort. In patients with habitual alcohol intake, however, pretreatment MCV showed the opposite prognostic role and pretreatment MCV positively correlated with the mortality and tumor recurrence after adjusting for other prognostic factors.

Previously, there were several studies focusing on the clinical role of MCV in CRC, showing MCV was associated with the risk of colorectal adenoma (10), advanced CRC (11) and response to chemotherapy in CRC (12, 13) with even conflicting results and only one study was available investigating the prognostic role of pretreatment MCV in CRC patients (16). In this study, Hidemasa and his colleagues carried out a retrospective analysis in 1174 patients with stage I, II, and III CRC, and it was found that MCV of <80 fL was a favorable prognostic factor in CRC. The opposite prognostic role in this study might result from the different patient populations included into the two studies, that early stage CRC were excluded from our analyses and the proportion of patients with habitual alcohol intake in our study might be different from it, with the finding that pretreatment MCV positively correlated with the mortality and tumor recurrence in patients with habitual alcohol intake.

**Figure 4:**
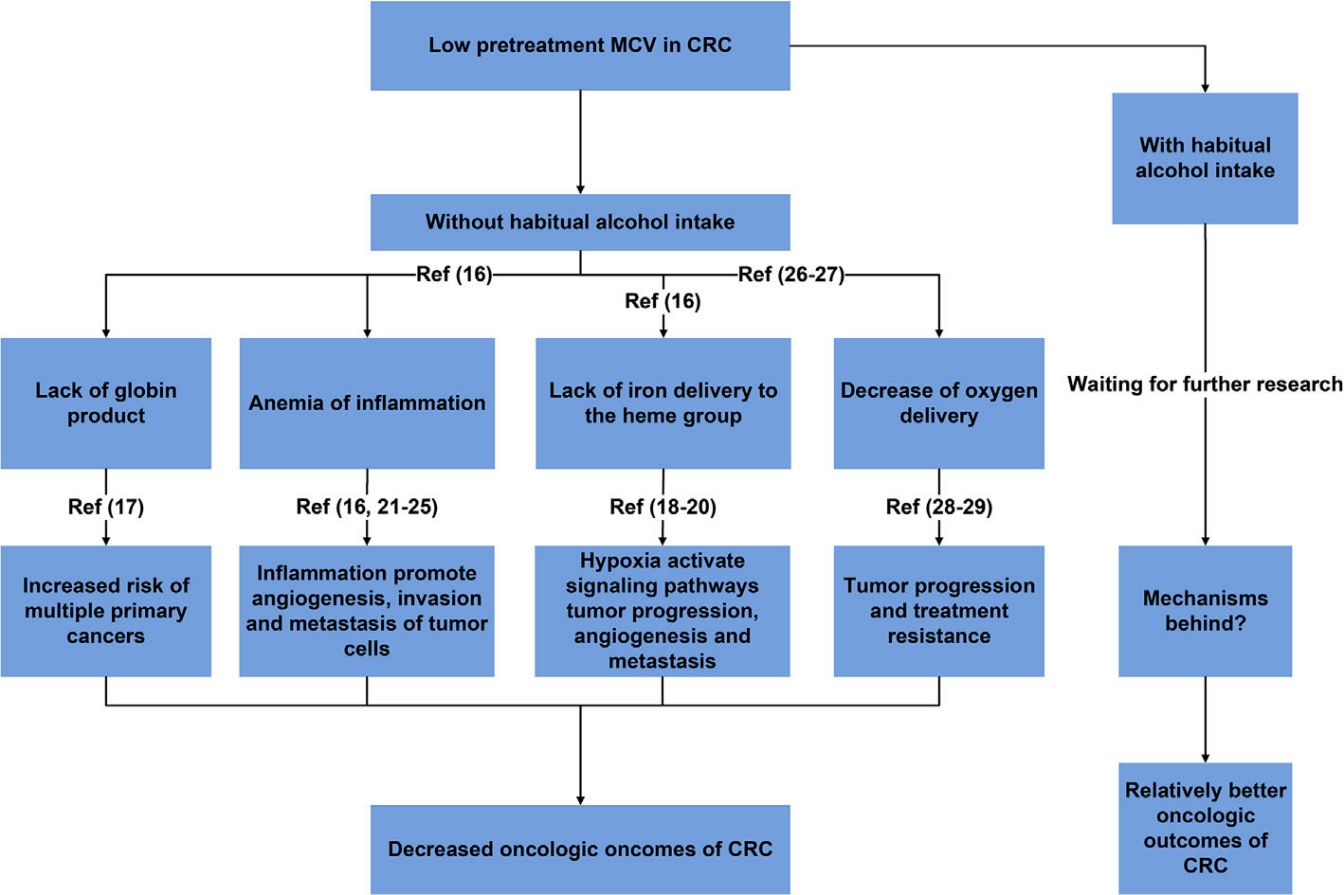
Summary of reasonable mechanisms behind the prognostic role of MCV with the implications of habitual alcohol intake.

Shown as **Figure 4**, reasonable mechanisms behind our findings were summarized. The decrease of pretreatment MCV might result from a lack of globin product (thalassemia), restricted iron delivery to the heme group of hemoglobin (anemia of inflammation) and a lack of iron delivery to the heme group (iron-deficiency anemia) (17). In 2015, Chung et al. (18) conducted a nationwide study of 2655 patients diagnosed with thalassemia between 1998 and 2010 by using data from the Taiwan Longitudinal Health Insurance Database with comparison to 10620 people without thalassemia from the general population and found that patients with thalassemia exhibited a 1.54-fold greater overall risk of cancer than the general population, meaning that lack of globin product would increase the risk of multiple primary cancers in addition to CRC.

Hypoxia, caused by iron deficiency anemia, would activate multicellular signaling pathways for cell survival, tumor progression, angiogenesis and metastasis. For example, hypoxia-hypoxiainducible-miR-210 would promote cell proliferation, vascular Endothelial Growth Factor (VEGF) expression and cell survival in hypoxic regions of tumors (19, 20). Moreover, immune system could also be affected by iron deficiency anemia, which decreased the proliferation and cytotoxic as well as phagocytic activities of the immune cells against tumor cells through downregulation of different immunological pathways, making patients with iron deficiency anemia more susceptible to development of cancer (21).

Inflammatory states were often associated microcytic anemia (17). As a response that an organism used to resolve infection, tissue injury or other cellular stress, and to restore tissue function through repair mechanism, inflammation also played an important role in cancers (22). Tumor associated inflammation was a source of survival, growth and pro-angiogenic factors, as well as extracellular matrix (ECM)-modifying enzymes that facilitate angiogenesis, invasion and metastasis of tumor cells (23, 24). Inflammation induced angiogenesis not only provided the necessary nutrients for tumor growth, but also provided a ‘highway’ for the tumor to escape from the primary tumor site to promote the distal metastasis of tumor cells. Inflammation could also suppress the anti-tumor immune responses, resulting into the escape of tumor cells from host immune surveillance, which was critical for almost all steps of metastatic tumor progression (25, 26).

In addition, it was found that higher MCV was associated with an elevated oxygen pressure (27) and an increased oxygen affinity in red blood cells (28). Then higher MCV could result in enhanced oxygen saturation in red blood cells. Therefore, higher MCV may facilitate oxygen delivery. Compared with normal MCV, decreased oxygen delivery in CRC with low MCV would result in decreased physical functions and hypoxia which played a main role in tumor progression and treatment resistance, then leading to worse oncologic outcomes (29, 30).

Alcoholism was a devastating disease which occurred in approximately 8% of the general population, and approximately 20% of hospitalized patients (31–35). Many previous researches supported a positive association between alcohol consumption and CRC risk (36–38). Our study also demonstrated that CRC patients with habitual alcohol intake was independently associated with 75.4% increased risk of overall mortality.

MCV had been reported to be increased in chronic alcoholism (39–42). We therefore investigated the prognostic role of pretreatment MCV in CRC patients with implications of habitual alcohol intake. To the best of our knowledge, it was the first study that showed pretreatment MCV positively correlated with the mortality and tumor recurrence in patients with habitual alcohol intake, indicating that researches focused on the clinical value of pretreatment MCV should take alcohol consumption status into consideration and earlier studies were thoughtless. However, mechanisms behind the opposite prognostic role of pretreatment MCV in CRC patients with habitual alcohol intake was still uncertain, and we believed our findings would elicit many fundamental studies to elucidate them.

There were a few limitations in the present study. First, this study was only a single-institution one, and the sample size was required to be enlarged. Second, some factors (including hypothyroidism, blood disease, liver disease and so on) which might affect the levels of pretreatment MCV were not taken into account in our analyses. Finally, the present study was only a retrospective one, and more evidence need to be provided by randomized controlled clinical trials to support our findings in the future.

## Conclusions

In CRC patients without habitual alcohol intake, low (<80 fL) level of pretreatment MCV was a predictor of poor prognosis. In patients with habitual alcohol intake, however, pretreatment MCV showed the opposite prognostic role and pretreatment MCV positively correlated with the mortality and tumor recurrence. We believed our findings would elicit many fundamental studies to elucidate the mechanisms behind.

## Data Availability Statement

The raw data supporting the conclusions of this article will be made available by the authors, without undue reservation.

## Author Contributions

XL and SZ conceptualized and designed the study. QL conducted the analyses of the study. QL and YY interpreted the data. QL drafted the manuscript. QL, XL, and SZ revised the manuscript. All authors contributed to the article and approved the submitted version.

## Funding

This research was supported by the National Natural Science Foundation of China (Nos. 81772599, 82002489 and 81972260) and Doctoral Entrepreneurship Project of Guangdong Provincial People's Hospital (Nos.2020bq19). The funders had no role in the study design, data collection and analysis, decision to publish, or preparation of the manuscript.

## Conflict of Interest

The authors declare that the research was conducted in the absence of any commercial or financial relationships that could be construed as a potential conflict of interest.

## Supplementary Material

Supplementary Figure 1 Flow chart of patient inclusion.

Supplementary Figure 2 Kaplan–Meier OS curves according to the levels of pretreatment MCV.
