# Supplementary figures and images for: Implications of Habitual Alcohol Intake With the Prognostic Significance of Mean Corpuscular Volume in Stage II-III Colorectal Cancer
Source: Front Oncol. 2021 Jun 14;11:681406. doi: 10.3389/fonc.2021.681406 (PMC8236820; doi:10.3389/fonc.2021.681406)

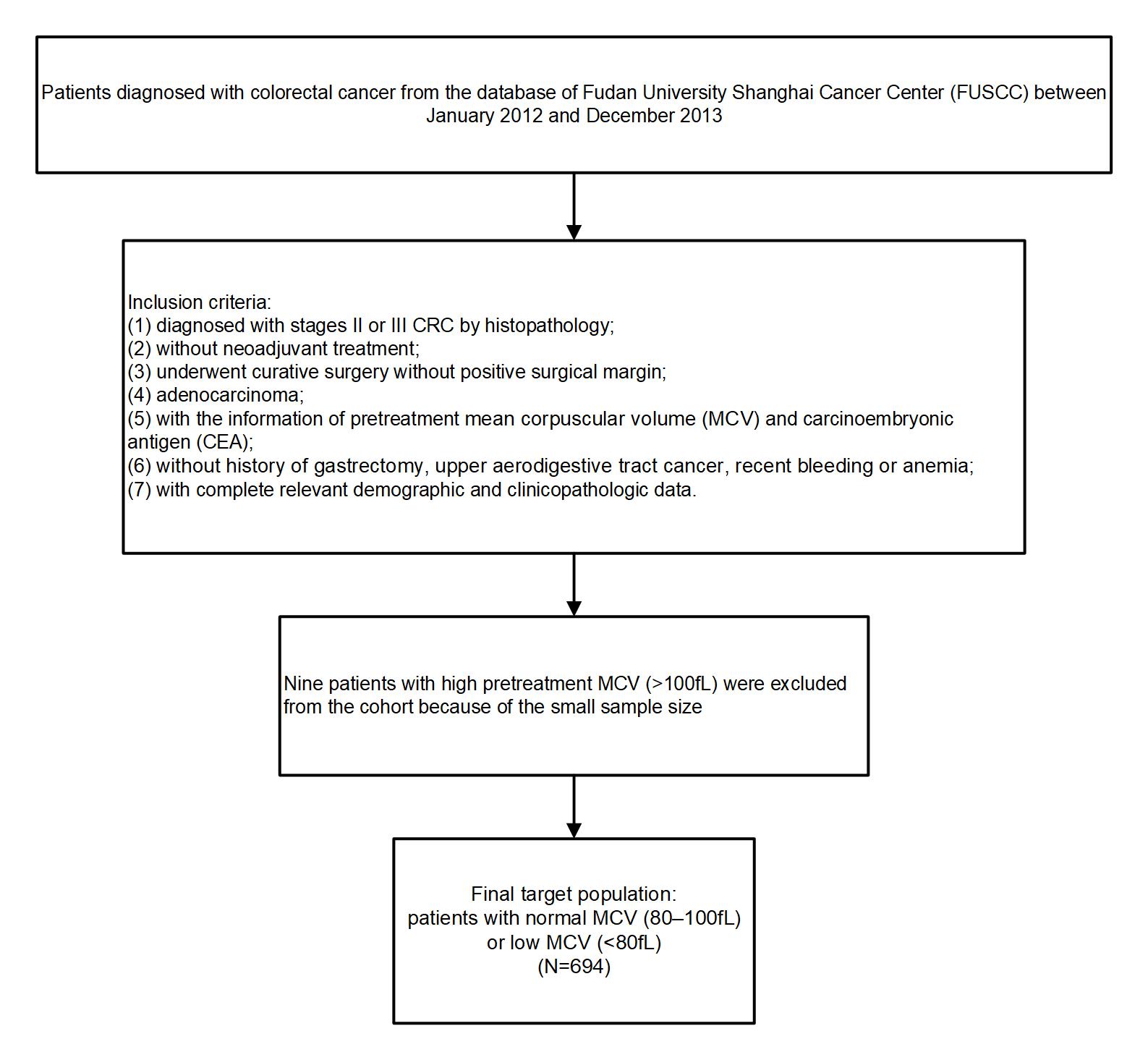

Supplement: Supplementary Figure 1 — Flow chart of patient inclusion. [file Image_1.jpeg]

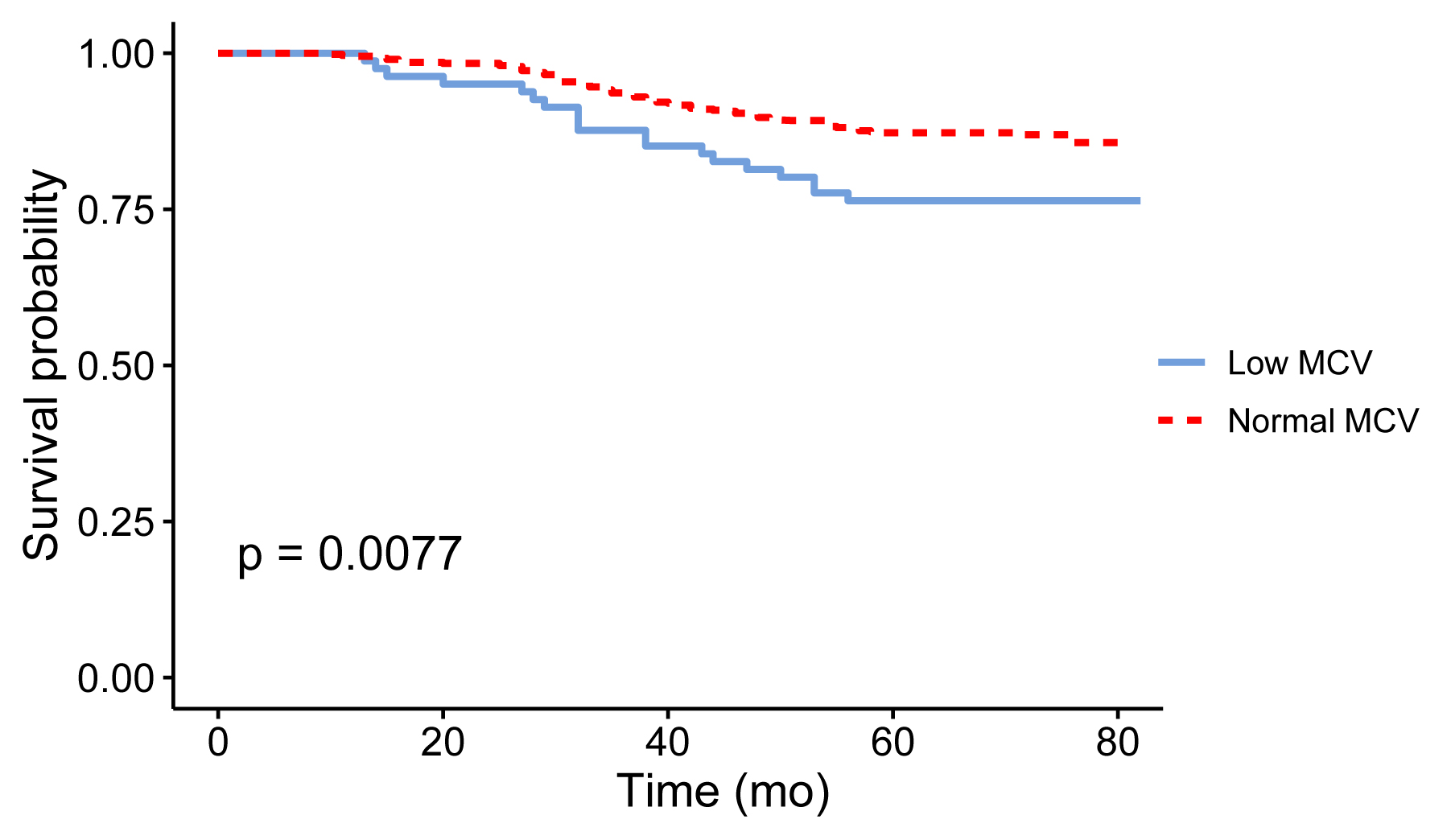

Supplement: Supplementary Figure 2 — Kaplan–Meier OS curves according to the levels of pretreatment MCV. [file Image_2.jpeg]
